# Direct Reprogramming of Somatic Skin Cells from a Patient with Huntington’s Disease into Striatal Neurons to Create Models of Pathology

**DOI:** 10.1134/S0012496623700849

**Published:** 2024-01-08

**Authors:** N. A. Kraskovskaya, M. G. Khotin, A. N. Tomilin, N. A. Mikhailova

**Affiliations:** Institute of Cytology, Russian Academy of Sciences, St. Petersburg, Russia

**Keywords:** direct reprogramming, Huntington’s disease, neurons, striatum, fibroblasts, huntingtin aggregates

## Abstract

A new in vitro model of Huntington’s disease (HD) was developed via a direct reprogramming of dermal fibroblasts from HD patients into striatal neurons. A reprogramming into induced pluripotent stem (iPS) cells is obviated in the case of direct reprogramming, which thus yields neurons that preserve the epigenetic information inherent in cells of a particular donor and, consequently, the age-associated disease phenotype. A main histopathological feature of HD was reproduced in the new model; i.e., aggregates of mutant huntingtin accumulated in striatal neurons derived from a patient’s fibroblasts. Experiments with cultured neurons obtained via direct reprogramming make it possible to individually assess the progression of neuropathology and to implement a personalized approach to choosing the treatment strategy and drugs for therapy. The in vitro model of HD can be used in preclinical drug studies.

Huntington’s disease (HD) is an incurable human genetic disease with dominant inheritance and is caused when the CAG codon expands beyond the threshold of 36 in the IT15 gene, which is also known as the HTT gene and codes for the protein hungtingtin [1]. The mean age at onset is 35–45 years, depending on the number of CAG repeats in the polymorphic gene locus. Repeat expansion leads to an elongated polyglutamine tract in the protein, and the mutant protein consequently aggregates in brain tissues [2]. Great achievements have been made with induced pluripotent stem (iPS) cells [3] and their derivative neurons [4–6]. However, several drawbacks are characteristic of iPS cells and substantially limit their application in modeling neurodegenerative disorders [7]. Passing through a pluripotent state, neuronal cells lose the majority of the epigenetic marks that their somatic progenitors have acquired during their growth and maturation [8]. Moreover, the extent of maturity in neurons produced through iPS cells strongly depends on the reprogramming protocol, which is often poorly reproducible [9].

Certain pathological alterations associated with neurodegenerative disorders, including HD, manifest themselves with age, when neurons undergo senescence and become more vulnerable to cell stress [10]. This aspect is important in the context of HD modeling, and we consequently focused on direct reprogramming as a new approach to modeling neuronal pathologies. A key feature is that the approach obviates dedifferentiation to an embryonic state, which is responsible for losses of epigenetic information inherent in a donor’s cells. The approach better preserves the age-associated disease phenotype [7, 11] and better reflects the characteristic neuronal phenotype (e.g., neuronal death) as compared with models where age-related epigenetic markers are absent. An age-specific gene transcription profile and a lower level of nuclear transport receptors are observed in neurons derived via direct reprogramming, but not in neurons derived via iPS cells [12]. Aging-related alterations characteristic of a donor are also better preserved in neurons derived via direct reprogramming, including DNA lesions, loss of heterochromatin and nuclear organization, and higher β-galactosidase activity [7].

Only four works have utilized direct reprogramming to study the HD pathogenesis to date [11, 13–15], mostly because a low efficiency is characteristic of the reprogramming procedure and a high heterogeneity is observed for neurons thus derived [16]. We have previously optimized the direct reprogramming protocol and thus increased both the survival of induced striatal neurons (up to 80%) and the homogeneity of the resulting cell population [17].

In this work, direct reprogramming was used to derive striatal neurons from the HDDF derval fibroblast line originating from a HD patient and the control DF1 line originating from a healthy donor [18]. Figure 1 shows the general appearance of in vitro cultures of dermal fibroblasts obtained from the HD patient and the donor, who had no neuronal pathology. Huntingtin showed a diffuse distribution through the cytoplasm without forming visible aggregates in both of the fibroblast populations (Fig. 2). However, a substantial difference was observed in striatal neurons derived from these fibroblasts. Huntingtin was still diffusely distributed through the cytoplasm and formed no visible aggregates in neurons derived from DF1 fibroblasts (Fig. 3). In contrast, huntingtin aggregates in the neuronal soma and some of the processes were observed in neurons derived from HDDF fibroblasts.

**Fig. 1.**
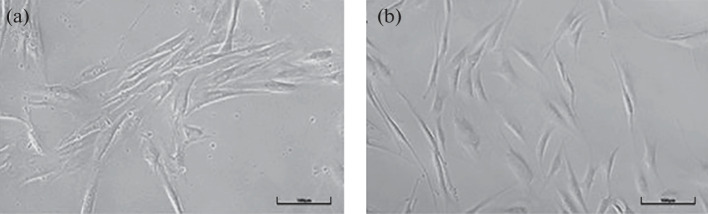
In vitro cultures of human dermal fibroblasts of the lines (a) DF1, which originates from a healthy donor, and (b) HDDF, which originates from a HD patient. Phase contrast microscopy, ×10. Bar, 100 µm.

**Fig. 2.**
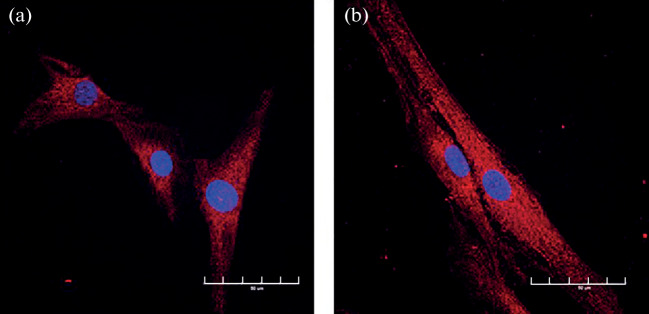
Diffuse distribution of huntingtin through the cytoplasm in dermal fibroblasts of the lines (a) DF1 from a healthy donor and (b) HDDF from a HD patient. Immunofluorescence staining with a mEM48 antibody to huntingtin (a secondary antibody conjugated with Alexa 555) (red). Cell nuclei were visualized using DAPI (blue). Confocal microscopy, ×60. Bar, 50 µm.

**Fig. 3.**
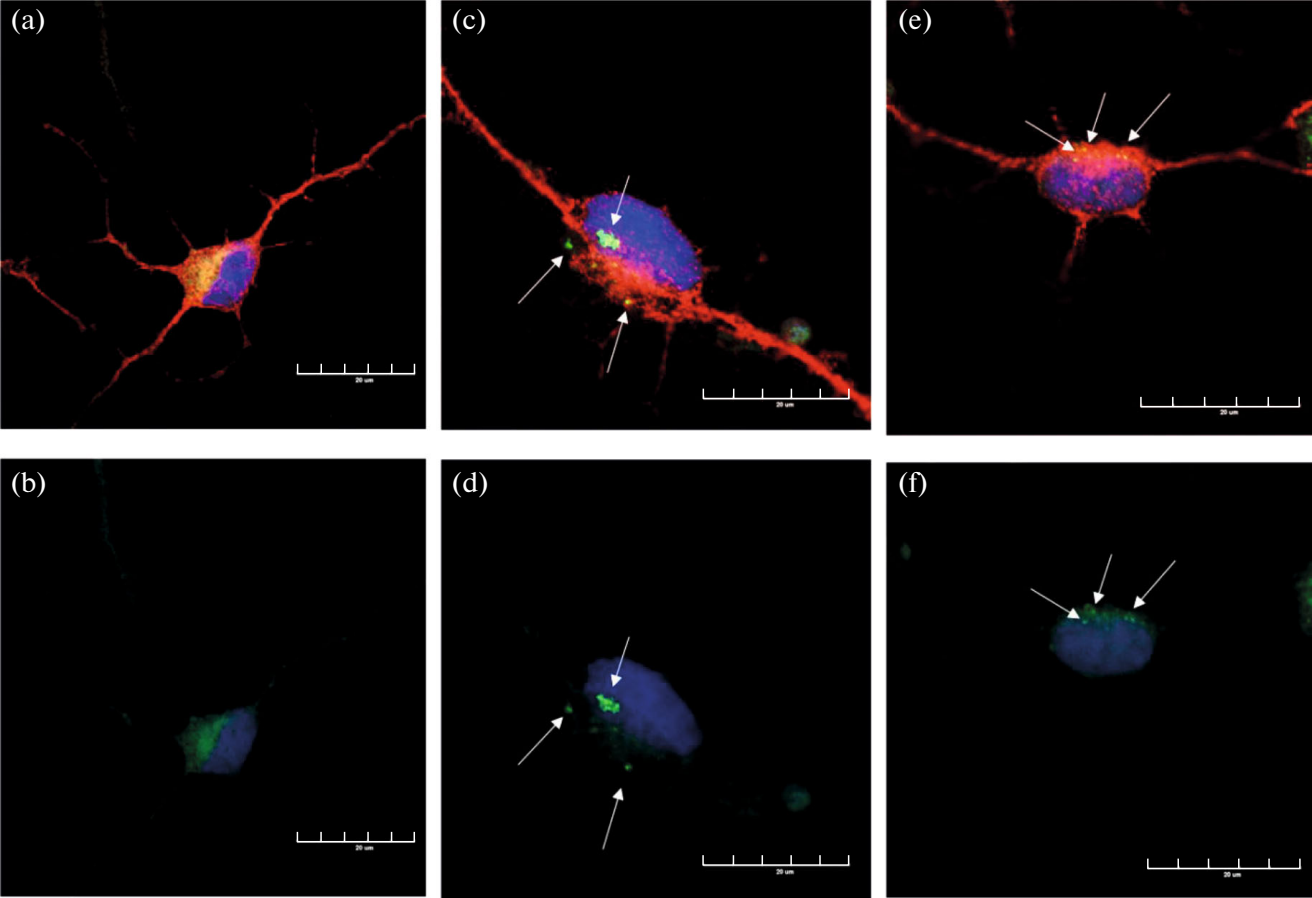
Huntingtin (green) visualization in striatal neurons derived from dermal fibroblasts via direct reprogramming. The fibroblast lines (a, b) DF1 from a healthy donor and (c–f) HDDF from a HD patient were used. Immunofluorescence staining with antibodies to the neuronal marker MAP2 (a secondary antibody conjugated with Alexa 555) and huntingtin (mEMM48, a secondary antibody conjugated with Alexa 488). Colocalization of the two proteins is seen as a yellow color. Cell nuclei were visualized using DAPI (blue). Confocal microscopy, ×60. Bar, 30 µm. (a, b) A diffuse huntingtin distribution is observed in striatal neurons derived from dermal fibroblasts of a healthy donor, while (c–f) huntingtin aggregates (arrows) are detectable in striatal neurons derived from dermal fibroblasts of a HD patient.

We thus confirmed that mutant protein aggregates, which are a key histopathological sign of HD, are observed in neurons derived from fibroblasts of HD patients. Our cell model reflects the main pathological alterations occurring in cells upon HD development and has a potential as a platform to evaluate the efficacies of potential drugs.

Current personalized approaches imply selection of effective medications for each individual patient. Although effective drugs to treat or prevent HD have not been developed as of yet, it is possible to assume that their efficacy depends on the patient’s genotype, age, and disease stage. Given that HD is an autosomal dominant disease, information on the specifics of HD course in the older generation of a family will help to develop therapies for HD carriers of younger generations with due regard to their age-related features. In this case, direct reprogramming provides an essential tool to choose the treatment that is most safe and effective for a particular patient.

## MATERIALS AND METHODS

**Isolation of dermal fibroblasts.** Fibroblasts of a patient with a verified diagnosis of HD were isolated from skin biopsy material according to a published protocol [18]. The patient was a 36-year-old woman with the established HD diagnosis. A genetic analysis revealed 47 CAG repeats in her huntingtin gene. Prior to collecting a tissue sample in a medical facility, the patient had a medical examination and provided her voluntary informed consent for tissue collection and use of derivative cells for scientific research. Experiments with cells were approved by the Ethics Committee of the Institute of Cytology, protocol no. 12/23 dated August 8, 2023.

Cells were mechanically isolated from a fragment of skin biopsy material and cultured in 90% DMEM/F12 (Biolot, Russia), 10% fetal bovine serum (Gibco, United States). Cells were passaged using 0.25% trypsin–0.2% Versen at 90% confluence. The split ratio was 1 : 3 to 1 : 5. Cells proliferated intensely, and their viability was 80% after cryopreservation. Dermal fibroblasts of the DF1 line were used as a control. The DF1 line was derived from a healthy donor, which was a 37-year-old woman (the line was obtained from the Vertebrate Cell Culture Collection of the Institute of Cytology).

**Direct reprogramming of dermal fibroblasts into striatal neurons.** The reprogramming procedure utilized the lentivirus vectors that coded for the microRNAs miR-9/9* and miR-124 (which initiate chromatin remodeling) and the transcription factors MYT1L, DLX2 (which direct differentiation into GABAergic neurons), and CTIP2 (which directs differentiation into striatal neurons). A doxycycline-inducible promoter was used to control microRNA expression. After antibiotic selection, postmitotic cells were seeded on Matrigel-coated glasses. The conditioned fibroblast medium was replaced on the next day with a reprogramming medium, which was the Neurobasal-Ас medium supplemented with 2% B-27, 0.125 mM GlutaMAX (all reagents were from Gibco, United States), 1 mM valproic acid, 1 µM retinoic acid, 200 µM dibutyryl-Camp (Sigma-Aldrich, United States), 20 ng/mL brain-derived neurotrophic factor, 20 mg/mL neurotrophin-3 (PeproTech, United Kingdom), and 20 ng/mL glial cell-derived neurotrophic factor. Cells were cultured for 40 days after infection with the lentiviruses. Huntingtin was visualized via immunofluorescence staining with a mEM48 antibody (Merck Millipore).
